# Supplementary material for: Research artifacts and citations in computer systems papers
Source: PeerJ Comput Sci. 2022 Feb 7;8:e887. doi: 10.7717/peerj-cs.887 (PMC9044204; doi:10.7717/peerj-cs.887)
Supplement: Supplemental Information 2 — Please refer to all *.md files for detailed data descriptions. [file peerj-cs-08-887-s002.tbz2 › sysconf/docs/index.html]

Statistical Observations on Systems Papers


- **1** Introduction
  - Research in Computer Systems
  - Result Highlights
  - Reproducibility
  - Acknowledgements
  - License
  - Citation
  - Version history
- **2** Data
  - **2.1** Conference data
    - **2.1.1** System conferences
    - **2.1.2** Additional conferences
    - **2.1.3** Conferece details
    - Field description
    - **2.1.4** Paper labels
  - **2.2** Person data
    - Field description
  - **2.3** Paper data
  - **2.4** Geographical data
  - **2.5** Challenges
    - **2.5.1** Defining “Systems”
    - **2.5.2** Author disambiguation
    - **2.5.3** Partial conference data
- **3** Features
  - **3.1** Conference-related variables
    - Field description
  - **3.2** Author-related variables
    - Field description
  - **3.3** Paper-related variables
  - **3.4** Textual-related variables
  - **3.5** Country-related variables
    - Field description
- Bibliography

# Statistical Observations on Systems Papers

# Statistical Observations on Systems Papers

*Eitan Frachtenberg*

*2019-12-20*

# 1 Introduction


What makes a research publication stand out?

What makes a journal or conference reputable?

What contributes to an author’s publication record?

Almost every scientist has been curious about questions like these at one time or another. And yet much to our frustration, we can’t answer them scientifically, because we don’t have accurate metrics to measure them by. And even if we did, we wouldn’t be able to measure them, because controlled experiments on the publication process are all but impossible.

Instead, what this document attempts to do is to address these questions through the observation of multi-dimensional data. The data has been collected from fifty well-known conferences in the computer systems field throughout 2017. It includes nearly every aspect of the publication process: conference statistics, author statistics, paper metrics, textual analysis, citation graphs, and many more.

The sheer volume and detail of the data reveal some interesting insights about publications in this specific area, and perhaps more broadly as well. The next section lists some of these high-level insights, while the rest of this document delves into the methods and the analysis in much greater detail. It starts with a precise description of both the complete data set that was collected in Chapter 2, and follows with a description of all derived metrics and variables (“features”) in Chapter 3. I suggest you at least skim both of these chapters to get an understanding of the data collected, and the possible relationships and questions that could arise from it.

The rest of the chapters each deal with a cohesive subset of questions. For example, Chapter **??** attempts to measure and compare various metrics of conference “quality”, while Chapter **??** does the same for individual papers. Although these chapters appear in sequence with later chapters sometimes referring to previous data, feel free to read these out of order or jump straight to the topic that interests you the most. You’ll find links to any prerequisite analysis from previous chapters.

## Research in Computer Systems

This study focuses almost exclusively on a single field of study, *computer systems*. Computer Systems (aka ‘*Systems*’) is the field that deals, in a nutshell, with building programs that use a lot of resources and profiling that resource usage. Computer systems is a large and rapidly evolving field in computer science. This field stands out from other areas of computer science in some key characteristics. It emphasizes scientific exploration through system implementation, and combines a unique blend of mathematical rigor, simulation science, and technical engineering.

Systems work includes building operating systems, computer architectures, databases, parallel and distributed computing, and networking among others. It is a highly practical field, focused on implementation and understanding what kinds of usage a system will be able to handle. As Craig Partridge writes [4]:

> “The classic systems paper presents an implementation or planned implementation. The implementation can be in software or hardware or both. The implementation’s contribution is usually that it either achieves some new function, never before achieved, or it realizes an existing function more efficiently or effectively than previously.”

Because of the applicability of systems work and its rapid pace of development, peer-reviewed conferences hold a special role in scholarly publication. System practitioners often prefer to publish their best work in a conference with a turn-around time of a few months, compared to a few years in some top systems journals [5]. Thus, conference publication takes the lead role in describing innovative research, while journals are often delegated to archival purposes [6]. This is why we chose to focus on the conference publication process in systems.

Specifically, we focus on an expansive, but nevertheless selective, cross-sectional set of systems conferences from 2017, all employing a rigorous peer-review process to select their papers (with typical acceptance rates in the vicinity of 20%). We collected statistics about every conference, every peer-reviewed paper in it, and every co-author of every paper. By combinining statistics from all these angles, we are able to explore hundreds of aspects of the field of computer systems, as reflected through its research papers.

## Result Highlights


Each chapter (starting from Ch. **??**) starts with a list of some of the interesting questions in the chapter. Feel free to skip around from chapter to chapter, review the highlights and questions, and then dive deeper into the details of interest to you.

## Reproducibility

Reproducibility and replication in research is a cornerstone of the scientific method. There’s an entire chapter (**??**) dedicated to reproducibility in computer systems here. Accordingly, this study also attempts to replicate or approximate numerous past results and assumptions about the publication and peer-review process (and refers to the original results when possible). These results not always be novel, but with reproducibility, that’s exactly the point!

It is only fitting then that we would also make every attempt to provide as much data and source code as necessary to reproduce all the results in this analysis [1]. The complete data, ETL scripts, and analysis code for this document can be found at https://github.com/eitanf/sysconf. This document (which itself includes the analysis source code) is included in the repository and is formatted with the Bookdown [8] and knitr [9] R packages.

Every data file or directory has a corresponding markdown file describing it. It resides in the same directory as the data file or sub-directory, and has the same name, with a .md extension.
The description file details the field descriptions and types, as well as the source script that generated it, the git hash of version that generated it, and the location in the source code that generated each field. This information is reproduced in the next two chapters.

The R analysis code embedded in this document ran with the following R session information:

```
## R version 3.4.4 (2018-03-15)
## Platform: x86_64-pc-linux-gnu (64-bit)
## Running under: Linux Mint 19.2
## 
## Matrix products: default
## BLAS: /usr/lib/x86_64-linux-gnu/blas/libblas.so.3.7.1
## LAPACK: /usr/lib/x86_64-linux-gnu/lapack/liblapack.so.3.7.1
## 
## locale:
##  [1] LC_CTYPE=en_US.UTF-8       LC_NUMERIC=C              
##  [3] LC_TIME=en_US.UTF-8        LC_COLLATE=en_US.UTF-8    
##  [5] LC_MONETARY=en_US.UTF-8    LC_MESSAGES=en_US.UTF-8   
##  [7] LC_PAPER=en_US.UTF-8       LC_NAME=C                 
##  [9] LC_ADDRESS=C               LC_TELEPHONE=C            
## [11] LC_MEASUREMENT=en_US.UTF-8 LC_IDENTIFICATION=C       
## 
## attached base packages:
## character(0)
## 
## other attached packages:
##  [1] bindrcpp_0.2.2        bookdown_0.14         cowplot_0.9.4        
##  [4] devtools_2.2.1        GGally_1.4.0          ggrepel_0.8.1        
##  [7] jsonlite_1.6          kableExtra_1.1.0      lubridate_1.7.4      
## [10] multidplyr_0.0.0.9000 plotly_4.9.0          psych_1.8.12         
## [13] purrr_0.3.2           readtext_0.75         reshape2_1.4.3       
## [16] rticles_0.11          textclean_0.9.3       textstem_0.1.4       
## [19] tidytext_0.2.2        tidyverse_1.2.1      
## 
## loaded via a namespace (and not attached):
##  [1] compiler_3.4.4  magrittr_1.5    graphics_3.4.4  htmltools_0.3.6
##  [5] tools_3.4.4     rstudioapi_0.10 utils_3.4.4     yaml_2.2.0     
##  [9] grDevices_3.4.4 Rcpp_1.0.2      stats_3.4.4     datasets_3.4.4 
## [13] stringi_1.4.3   rmarkdown_1.16  knitr_1.25      methods_3.4.4  
## [17] stringr_1.4.0   digest_0.6.21   xfun_0.10       base_3.4.4     
## [21] evaluate_0.14
```

## Acknowledgements

## License

Copyright 2017–2019. This work is licensed under CC-BY-SA 4.0.

## Citation

To cite this report, please use the following BibTeX entry:

```
@Report{frachtenberg19:sysconf,
  author =   {Eitan Frachtenberg},
  title =    {Statistical Observations on Systems Papers},
  year =     2019,
  url =      {http://github.com/eitanf/sysconf/},
  note =     {Version 2019-12-18}
}
```

## Version history

The release history of this document can be found in the Release notes

### Bibliography

[1] Christopher, G. 2013. *Reproducible research with R and RStudio*. CRC Press.

[4] Partridge, C. 1998. How to increase the chances your paper is accepted at ACM SIGCOMM. *COMPUTER COMMUNICATION REVIEW*. 28, (1998), 70–74.

[5] Patterson, D., Snyder, L. and Ullman, J. 1999. Evaluating computer scientists and engineers for promotion and tenure. *Computing Research News*. (1999).

[6] Vrettas, G. and Sanderson, M. 2015. Conferences versus journals in computer science. *Journal of the Association for Information Science and Technology*. 66, 12 (2015), 2674–2684.

[8] Xie, Y. 2016. *Bookdown: Authoring books and technical documents with R markdown*. CRC Press.

[9] Xie, Y. 2014. Knitr: A comprehensive tool for reproducible research in R. *Implementing reproducible computational research*. V. Stodden, F. Leisch, and R.D. Peng, eds. Chapman; Hall/CRC.
