# Supplementary material for: Research artifacts and citations in computer systems papers
Source: PeerJ Comput Sci. 2022 Feb 7;8:e887. doi: 10.7717/peerj-cs.887 (PMC9044204; doi:10.7717/peerj-cs.887)
Supplement: Supplemental Information 2 — Please refer to all *.md files for detailed data descriptions. [file peerj-cs-08-887-s002.tbz2 › sysconf/docs/references.html]

Bibliography | Statistical Observations on Systems Papers


- **1** Introduction
  - Research in Computer Systems
  - Result Highlights
  - Reproducibility
  - Acknowledgements
  - License
  - Citation
  - Version history
- **2** Data
  - **2.1** Conference data
    - **2.1.1** System conferences
    - **2.1.2** Additional conferences
    - **2.1.3** Conferece details
    - Field description
    - **2.1.4** Paper labels
  - **2.2** Person data
    - Field description
  - **2.3** Paper data
  - **2.4** Geographical data
  - **2.5** Challenges
    - **2.5.1** Defining “Systems”
    - **2.5.2** Author disambiguation
    - **2.5.3** Partial conference data
- **3** Features
  - **3.1** Conference-related variables
    - Field description
  - **3.2** Author-related variables
    - Field description
  - **3.3** Paper-related variables
  - **3.4** Textual-related variables
  - **3.5** Country-related variables
    - Field description
- Bibliography

# Statistical Observations on Systems Papers

# Bibliography

[1] Christopher, G. 2013. *Reproducible research with R and RStudio*. CRC Press.

[2] Haak, L.L., Fenner, M., Paglione, L., Pentz, E. and Ratner, H. 2012. ORCID: A system to uniquely identify researchers. *Learned Publishing*. 25, 4 (2012), 259–264.

[3] Han, H., Giles, L., Zha, H., Li, C. and Tsioutsiouliklis, K. 2004. Two supervised learning approaches for name disambiguation in author citations. *Proceedings of the 2004 joint ACM/IEEE conference on digital libraries* (2004), 296–305.

[4] Partridge, C. 1998. How to increase the chances your paper is accepted at ACM SIGCOMM. *COMPUTER COMMUNICATION REVIEW*. 28, (1998), 70–74.

[5] Patterson, D., Snyder, L. and Ullman, J. 1999. Evaluating computer scientists and engineers for promotion and tenure. *Computing Research News*. (1999).

[6] Vrettas, G. and Sanderson, M. 2015. Conferences versus journals in computer science. *Journal of the Association for Information Science and Technology*. 66, 12 (2015), 2674–2684.

[7] Wickham, H. 2014. Tidy data. *Journal of Statistical Software*. 59, 10 (2014), 1–23.

[8] Xie, Y. 2016. *Bookdown: Authoring books and technical documents with R markdown*. CRC Press.

[9] Xie, Y. 2014. Knitr: A comprehensive tool for reproducible research in R. *Implementing reproducible computational research*. V. Stodden, F. Leisch, and R.D. Peng, eds. Chapman; Hall/CRC.
