# Supplementary material for: Research artifacts and citations in computer systems papers
Source: PeerJ Comput Sci. 2022 Feb 7;8:e887. doi: 10.7717/peerj-cs.887 (PMC9044204; doi:10.7717/peerj-cs.887)
Supplement: Supplemental Information 2 — Please refer to all *.md files for detailed data descriptions. [file peerj-cs-08-887-s002.tbz2 › sysconf/docs/features.html]

3 Features | Statistical Observations on Systems Papers


- **1** Introduction
  - Research in Computer Systems
  - Result Highlights
  - Reproducibility
  - Acknowledgements
  - License
  - Citation
  - Version history
- **2** Data
  - **2.1** Conference data
    - **2.1.1** System conferences
    - **2.1.2** Additional conferences
    - **2.1.3** Conferece details
    - Field description
    - **2.1.4** Paper labels
  - **2.2** Person data
    - Field description
  - **2.3** Paper data
  - **2.4** Geographical data
  - **2.5** Challenges
    - **2.5.1** Defining “Systems”
    - **2.5.2** Author disambiguation
    - **2.5.3** Partial conference data
- **3** Features
  - **3.1** Conference-related variables
    - Field description
  - **3.2** Author-related variables
    - Field description
  - **3.3** Paper-related variables
  - **3.4** Textual-related variables
  - **3.5** Country-related variables
    - Field description
- Bibliography

# Statistical Observations on Systems Papers

# 3 Features

Many of the raw data fields described in the previous section are also used as variables in the statistical models that follow in subsequent chapters. Additional variables (some predictors, some dependent, and some both) were derived from the raw data files. The complete set of variables (and their derivation) is described in this chapter.

The feature files are all in tidy CSV format [7], under the `features/` sub-directory. The three primary files, `confs.csv`, `persons.csv`, and `papers.csv` roughly correspond to (and aggregate) their counterparts described in the previous chapter. But they also contain blended features computed by combining data from multiple sources. These tables can be joined by their key field (typically the first column of each file).

Some of these variables are so-called dummy variables. They convert a variable type from a categorical enumeration to a set of Boolean values. For example, the data field `organization` in the `data/conf/` conference files can take on one or more of the values “IEEE”, “ACM”, or “USENIX”. In the `data/features/all_confs.dat` file, this variable is split into three Boolean variables: `is_org_IEEE`, `is_org_ACM`, `is_org_USENIX`.

## 3.1 Conference-related variables

The file `confs.csv` contains features and aggregated statistics relating to each conference in the set.
The data was generated by `/data/sdb/Dropbox/sysconf/src/gather_confs.py` from git hash de05c66

### Field description

- `conference` (string): Short name of the conference (unique).
  - `is_org_ACM` (bool): Conference sponsored/organized by ACM.
  - `is_org_IEEE` (bool): Conference sponsored/organized by IEEE.
  - `is_org_USENIX` (bool): Conference sponsored/organized by USENIX.
  - `field` (category): The top-level category of the conference’s topic.
  - `subfield` (category): The top-ocurring topic of interest for this conference.
  - `url` (string): The URL of the main web page for this conference.
  - `country` (category): Two-letter name of country when conference took place.
  - `postdate` (date): First day of conference.
  - `month_of_year` (int): Month of year (1-12) of the conference’s postdate.
  - `months_since_published` (int): Months passed since the postdate to date of feature extraction.
  - `deadline_day_of_week` (category): The day of week of the last submission deadline (3-letter categorical abberviation).
  - `review_days` (int): The number of days between full paper submission due date and author notification.
  - `mean_pages` (numeric): Average number of pages in PDF version of accepted papers..
  - `submissions` (int): Total number of papers submitted for review.
  - `min_reviews` (int): The minimum number of reviews received by each paper.
  - `total_reviews` (int): The total number of formal reviews written by the PC, overall.
  - `double_blind` (bool): Whether the review process was double-blind.
  - `rebuttal` (bool): Were authors afforded an opportunity to answer the reviews before final acceptance decision was made?.
  - `open_access` (bool): Whether conference is open access.
  - `diversity_effort` (bool): Did the conference explicitly attempt to increase diversity?.
  - `age` (int): Approximate age (in years) of this conference series.
  - `past_papers` (int): How many papers were published in this series prior to publication year.
  - `mean_historical_length` (number): Average number of papers per conference (in the series) for previous years.
  - `past_citations` (int): How many total citations have papers in this series received, at approximately the postdate.
  - `mean_historical_citations` (number): Average number of citations per paper in past conferences in the series.
  - `h5_index` (int): The H-index of the conference in the 5 years preceding the postdate.
  - `h5_median` (number): The median H-index per paper in the conference in the 5 years preceding the postdate.
  - `chairs_num` (int): The number of program committee chairs.
  - `pc_size` (int): Number of technical PC members.
  - `pc_author_ratio` (number): Average number of PC members per unique author.
  - `npapers` (int): How many research papers were published in the proceedings.
  - `authors_num` (int): Total number of unique authors.
  - `mean_authors_per_paper` (number): Average number of co-authors per paper.
  - `acceptance_rate` (number): Ratio between number of accepted papers and number of submitted papers.
  - `pc_paper_ratio` (number): Ratio of papers (out of 100%) that have at least one author who is a PC member.

## 3.2 Author-related variables

The file `persons.csv` contains aggregated information about all authors, TPC chairs, and other roles in the selected conference subset.
The data was generated by `/data/sdb/Dropbox/sysconf/src/gather_persons.py` from git hash de05c66

### Field description

- `name` (string): Full person name, normalized and quoted.
  - `gs_email` (string): The email affiliation of the author as reported by GS (latest).
  - `gender` (categorical string): Verified or inferred gender.
  - `country` (categorical string): Two-letter country code from email affiliation (either paper or GS).
  - `sector` (categorical string): Employer sector from email affiliation (either paper or GS).
  - `npubs` (int): Author’s total publications (minimum across all conferences).
  - `hindex` (int): Author’s H-index (minimum).
  - `hindex5y` (int): Author’s H-index for past 5 years (minimum).
  - `i10index` (int): Author’s i10 index (minimum).
  - `i10index5y` (int): Author’s i10 index for past 5 years (minimum).
  - `citedby` (int): Author’s total citations (minimum).

## 3.3 Paper-related variables

- award: Whether a paper won an award at the conference (Boolean).
- title\_length: How many words are in the paper’s title?
  has\_subtitle: Does the paper title include a subtitle? (usually denoted by a colon or an em-dash)
- title\_colon: Does the paper have a single word followed by a colon in its title? Many systems papers introduce a new implemented systems and their titles are of the format: “Gizmo: an amazing new system to do something efficiently”. Some have the rarer format: “main title string: sub-title string”. This Boolean captures both forms.
- is\_topic\_\*: Dummy variables (Booleans) for the various topics a paper can discuss.
- months\_to\_gs: How many months (rounded down) did it take for GS to show a record for the paper?
- months\_to\_eprint: How many months (rounded down) did it take for GS to show a link to a downloadable version of the paper?

## 3.4 Textual-related variables

The full-text papers in PDF were first converted to textual format using pdftotext (v. 0.41.0), a utility included in the Poppler package. In rare instances, the paper’s text was embedded as an image, which required text extraction using the Tesseract optical character recognition package. The wrapper for this conversion can be found in `src/pdfocr.py`.

Each of these text files in turn was converted to “bag-of-words” format, which is simply a mapping from words to word counts. The output of this process is one CSV file per paper, each with two columns, one for normalized words, and one for the number of time each normalized word appeared in the paper. These data files are part of the accompanying data set, and can be found in the the `features/bow/` sub-directory.

The normalization of words is a process (coded in `src/normalize_text.py`) that includes the following stages:

- lower-case
  - lemmatization

## 3.5 Country-related variables

### Field description

- `code`: The two-letter international code of the country (also the top-level domain for the country).
  - `name`: String of country name.
  - `region`: String of geographical region or continent of the country.
  - `subregion`: String of geographical subregion.
  - `timezone` timezone as different in hours from GMT (at capital city, if more than one).
  - `speaks_english`: Boolean of whether English is one of the official languages in the country.

### Bibliography

[7] Wickham, H. 2014. Tidy data. *Journal of Statistical Software*. 59, 10 (2014), 1–23.
