# Supplementary material for: Research artifacts and citations in computer systems papers
Source: PeerJ Comput Sci. 2022 Feb 7;8:e887. doi: 10.7717/peerj-cs.887 (PMC9044204; doi:10.7717/peerj-cs.887)
Supplement: Supplemental Information 2 — Please refer to all *.md files for detailed data descriptions. [file peerj-cs-08-887-s002.tbz2 › sysconf/docs/data.html]

2 Data | Statistical Observations on Systems Papers


- **1** Introduction
  - Research in Computer Systems
  - Result Highlights
  - Reproducibility
  - Acknowledgements
  - License
  - Citation
  - Version history
- **2** Data
  - **2.1** Conference data
    - **2.1.1** System conferences
    - **2.1.2** Additional conferences
    - **2.1.3** Conferece details
    - Field description
    - **2.1.4** Paper labels
  - **2.2** Person data
    - Field description
  - **2.3** Paper data
  - **2.4** Geographical data
  - **2.5** Challenges
    - **2.5.1** Defining “Systems”
    - **2.5.2** Author disambiguation
    - **2.5.3** Partial conference data
- **3** Features
  - **3.1** Conference-related variables
    - Field description
  - **3.2** Author-related variables
    - Field description
  - **3.3** Paper-related variables
  - **3.4** Textual-related variables
  - **3.5** Country-related variables
    - Field description
- Bibliography

# Statistical Observations on Systems Papers

# 2 Data

> “In God we trust, all others must bring data.” – W. Edwards Deming

This chapter describes the main raw data collected, and the format of the files containing the data. All of these files reside in the `data/` directory and sub-directories in the repository. Some data files that are specific to a single chapter are described in that chapter later on.

## 2.1 Conference data

### 2.1.1 System conferences

Fifty Conferences in the systems areas were chosen as a data source. These conferences were selected to represent a wide range of venues and topics in computer systems during 2017, ranging from small workshops to week-long conferences with over 100 papers. This selection was not meant to be exhaustive, and doesn’t necessarily reflect the ``best’’ conferences in the field. But all conferences were peer-reviewed and sponsored by major organizations such as IEEE, ACM, and USENIX. (For conferences with multiple tracks, such as research and industry, only the peer-reviewed, full-paper tracks were considered.)

The full list of systems conferences can be found in the file `data/all_confs.json` and is reproduced in Table 2.1. One representative topic was chosen for each conference as described in Sec. **??**.

Table 2.1: Systems conferences used for this data set

| Name | Commencement | Topic | Papers | URL |
| --- | --- | --- | --- | --- |
| CIDR\_17 | 2017-01-08 | Data | 32 | http://cidrdb.org/cidr2017/ |
| PPoPP\_17 | 2017-02-04 | Concurrency | 29 | http://ppopp17.sigplan.org/ |
| HPCA\_17 | 2017-02-04 | Architecture | 50 | http://hpca2017.org |
| NDSS\_17 | 2017-02-26 | Security | 68 | https://www.ndss-symposium.org/ndss2017/ |
| FAST\_17 | 2017-02-27 | Storage | 27 | https://www.usenix.org/conference/fast17/ |
| NSDI\_17 | 2017-03-27 | Network | 42 | https://www.usenix.org/conference/nsdi17/ |
| ASPLOS\_17 | 2017-04-08 | Architecture | 56 | http://novel.ict.ac.cn/ASPLOS2017/ |
| VEE\_17 | 2017-04-09 | VM | 18 | http://conf.researchr.org/home/vee-2017 |
| ICPE\_17 | 2017-04-22 | Benchmark | 29 | https://icpe2017.spec.org/ |
| ISPASS\_17 | 2017-04-24 | Benchmark | 24 | http://www.ispass.org/ispass2017/ |
| EuroSys\_17 | 2017-04-23 | Concurrency | 41 | https://eurosys2017.github.io/ |
| HotOS\_17 | 2017-05-07 | OS | 29 | https://www.sigops.org/hotos/hotos17/ |
| CCGrid\_17 | 2017-05-14 | Cloud | 72 | https://www.arcos.inf.uc3m.es/wp/ccgrid2017/ |
| SIGMOD\_17 | 2017-05-14 | DB | 96 | http://sigmod2017.org/ |
| PODS\_17 | 2017-05-14 | DB | 29 | http://sigmod2017.org/pods-program/ |
| SYSTOR\_17 | 2017-05-22 | Storage | 16 | https://www.systor.org/2017/ |
| HCW\_17 | 2017-05-29 | GPGPU | 7 | http://hcw.eecs.wsu.edu/ |
| IPDPS\_17 | 2017-05-29 | Concurrency | 116 | http://www.ipdps.org/ipdps2017/ |
| SIGMETRICS\_17 | 2017-06-05 | Benchmark | 27 | http://www.sigmetrics.org/sigmetrics2017 |
| ISC\_17 | 2017-06-18 | HPC | 22 | http://isc-hpc.com/id-2017.html |
| ISCA\_17 | 2017-06-24 | Architecture | 54 | http://isca17.ece.utoronto.ca/doku.php |
| CLOUD\_17 | 2017-06-25 | Cloud | 29 | http://www.thecloudcomputing.org/2017/ |
| HPDC\_17 | 2017-06-28 | HPC | 19 | http://www.hpdc.org/2017/ |
| HotCloud\_17 | 2017-07-10 | Cloud | 19 | https://www.usenix.org/conference/hotcloud17 |
| HotStorage\_17 | 2017-07-10 | Storage | 21 | https://www.usenix.org/conference/hotstorage17 |
| ATC\_17 | 2017-07-12 | OS | 60 | https://www.usenix.org/conference/atc17 |
| SPAA\_17 | 2017-07-24 | Concurrency | 31 | http://spaa.acm.org/2017/index.html |
| PODC\_17 | 2017-07-25 | Concurrency | 38 | https://www.podc.org/podc2017/ |
| ICPP\_17 | 2017-08-14 | Concurrency | 60 | http://www.icpp-conf.org/2017/index.php |
| SIGCOMM\_17 | 2017-08-21 | Network | 36 | http://conferences.sigcomm.org/sigcomm/2017/ |
| EuroPar\_17 | 2017-08-30 | Concurrency | 50 | http://europar2017.usc.es/ |
| Cluster\_17 | 2017-09-05 | HPC | 65 | https://cluster17.github.io/ |
| PACT\_17 | 2017-09-11 | Architecture | 25 | https://parasol.tamu.edu/pact17/ |
| MASCOTS\_17 | 2017-09-20 | Benchmark | 20 | http://mascots2017.cs.ucalgary.ca/ |
| SOCC\_17 | 2017-09-25 | Cloud | 45 | https://acmsocc.github.io/2017/ |
| IISWC\_17 | 2017-10-02 | Benchmark | 31 | http://www.iiswc.org/iiswc2017/index.html |
| SP\_17 | 2017-05-22 | Security | 60 | https://www.ieee-security.org/TC/SP2017/index.html |
| MICRO\_17 | 2017-10-16 | Architecture | 61 | https://www.microarch.org/micro50/ |
| MobiCom\_17 | 2017-10-17 | Network | 35 | https://sigmobile.org/mobicom/2017/ |
| IGSC\_17 | 2017-10-23 | Energy | 23 | http://igsc.eecs.wsu.edu/ |
| ICAC\_17 | 2017-07-18 | OS | 14 | http://icac2017.ece.ohio-state.edu/ |
| CCS\_17 | 2017-10-31 | Security | 151 | https://www.sigsac.org/ccs/CCS2017/ |
| SOSP\_17 | 2017-10-29 | OS | 39 | https://www.sigops.org/sosp/sosp17/ |
| IMC\_17 | 2017-11-01 | Benchmark | 28 | http://conferences.sigcomm.org/imc/2017/ |
| SC\_17 | 2017-11-14 | HPC | 61 | http://sc17.supercomputing.org/ |
| Middleware\_17 | 2017-12-11 | OS | 20 | http://2017.middleware-conference.org/ |
| CoNEXT\_17 | 2017-12-13 | Network | 32 | http://conferences2.sigcomm.org/co-next/2017/#!/home |
| HiPC\_17 | 2017-12-18 | Concurrency | 41 | http://hipc.org/ |
| HPCC\_17 | 2017-12-18 | HPC | 77 | http://hpcl.seas.gwu.edu/hpcc2017/ |
| HotI\_17 | 2017-08-28 | Network | 13 | http://www.hoti.org/hoti25/archives/ |

… Some of these conferences may arguably be exluded from the “systems” label. For example (copy discussion about SPAA, etc.). But as we find in Ch. [graph analysis], there are strong relationships: some of these authors publish in “systemy” conferences, or collaborate with their authors, or cite their papers…

### 2.1.2 Additional conferences

In addition to the fifty systems conferences, six other conferences in related (but separate) fields were selected for contrast and comparison: three on programming languages and three on data mining/data science/information retrieval, as shown in Table 2.2

Table 2.2: Non-systems conferences used for this data set

| Name | Commencement | Papers | URL |
| --- | --- | --- | --- |
| OOPSLA\_17 | 2017-10-25 | 66 | https://2017.splashcon.org/track/splash-2017-OOPSLA |
| PLDI\_17 | 2017-06-18 | 47 | http://pldi17.sigplan.org/home |
| SLE\_17 | 2017-10-23 | 24 | http://www.sleconf.org/2017/ |
| ICDM\_17 | 2017-11-19 | 72 | http://icdm2017.bigke.org/ |
| KDD\_17 | 2017-08-15 | 64 | http://www.kdd.org/kdd2017/ |
| SIGIR\_17 | 2017-08-07 | 78 | http://sigir.org/sigir2017/ |

Table: (#tab:other-confs) Non-systems conferences used for this data set.

### 2.1.3 Conferece details

Each conference is described in its own data file. Most of the data was manually retrieved from the conference web sites (from the URLs in the previous tables)). Some information was personally requested from the TPC chair or obtained as described in the specific field’s description. Additionally, all the papers in the collection were downloaded in PDF format via open access or through digital libraries.1 The manual assignment of topics and content tags to papers, as described below, was accomplished by reviewing each of these papers.

The JSON files under the `data/conf/` directory hold information about each conference (one per file, with the filename being the short conference name). The data was copied from the conferences’ web pages, with supplemental information from the CFP, the proceedings and its frontmatter, and sometimes directory from program chairs.

Empty fields (where not data was available or applicable) are denoted as empty strings or lists, according to the field’s type.

### Field description

- `key` (string): Short conference name.
- `conference` (string): Full conference name.
- `url` (string): the URL of the main page for the conference.
- `ogranization` (list of categorical strings): List of organizations sponsonring the event (only IEEE/ACM/USENIX are recorded since the rest are too infrequent to provide useful signal).
- `country` (two-letter categorical string): Country code where conference took place.
- `postdate` (date): First day of technical program.
- `last_deadline` (date): The last date when author could upload the full version of their paper for review (before rebuttals and revisions).
- `review_days` (int): The number of days between full paper submission due date and author notification.
- `mean_pages` (numeric): Average number of pages in the PDF versions of accepted papers.
- `submissions` (int): Total number of papers submitted for review. Papers rejected outright for formatting violations are not counted.
- `min_reviews` (int): The minimum number of reviews received by each paper.
- `total_reviews` (int): The total number of formal reviews written by the PC, overall.
- `double_blind` (bool): Whether the review process was double-blind, i.e., author identities were hidden.
- `rebuttal` (bool): Were authors afforded an opportunity to answer the reviews before final acceptance decision was made?
- `open_access` (bool): Whether conference is open access. Are all papers freely available to download?
- `age` (int): Approximate age (in years) of this conference series (trying to account for splits and mergers).
- `past_papers` (int): How many papers were published in this series prior to 2017. This datum was either taken from the ACM digital library, IEEE Xplore, or counted manually.
- `past_citations` (int): How many total citations have papers in this series received, at approximately the postdate. This information was also copied from ACM digital library or IEEE Xplore, where available. Note, however, that because of differences in methods of citation counting, they are likely not directly comparable to each other or to Google Scholar’s count.
- `h5_index` (int): The “H-index” of the conference in the past 5 years, taken from Google Scholar Metrics, where available, at approximately the postdate.
- `h5_median (int)`: The median number of citations for papers in the h5 set, taken from Google Scholar Metrics, where available, at approximately the postdate.
- `field` (categorical string): The top-level category of the conference’s topic.
- `subfield` categorical string): The top-ocurring topic of interest for this conference (selected from `topics.json` for systems conferences).
- `diversity_effort` (bool): Did the conference website describe some effort to improve diversity (with details in the `notes` field).
- `notes` (string): free-form text with comments on the review proccess, if applicatible.
- `pc_chairs` (list of strings): Name(s) of the Program Committe (PC) chairs and their affiliations
- `pc_members` (list of strings): Names of the PC members and their affiliations.
- `keynote_speakers` (list of strings): Names of keynote (or invited) speakers.
- `session_chairs` (list of strings): Names of session chairs copied from the program (with repeats).
- `panelists` (list of strings): Names of panelists and moderators of panels.
- papers (list of dictionaries): Accepted peer-reviewed papers. For each paper, the following data were recorded:
  - `key` (string): A unique identifier combining the conference name and the paper’s ordinal number.
  - `title` (string): Paper title, copied from the conference web page
  - `authors` (list of strings): Author names, and sometimes affiliation in parenthesis. The affiliation wasn’t used for any features or training, but rather to help disambiguate among multiple scholar profiles with the same name, as described in the next section.
  - `award` (bool): Did the paper receive an award, such as “Best Paper” or “Best Student Paper”?
  - `artifact` (bool): Did the paper receive an “artifacts evaluated”, “artifacts available” or similar seal? (primarily ACM papers; see https://www.acm.org/publications/policies/artifact-review-badging)
  - `s2pid` (hexadecimal string)`: An identifier for the paper in semanticscholar.org.
  - `words` (int): Approximate number of words in the paper (wc of text file).
  - `topics` (list of categorical strings): List of keywords (see `topics.csv` for full list). These are high-level topics that roughly correspond to undergraduate courses in computer systems, such as “Operating Systems”, “Networking”, or “Security”. They were manually assigned, and as such, likely include some errors, misrepresentations, and ommisions. Each topic had at least one conference focused primarily on this topic, although multi-topic papers are common. The following table lists the topic selection that was used.
  - `content_tags` (dictionary of lists of categorical strings): List of research categories for the paper, taken from `data/content_tags.json` (currently all are manually assigned).

### 2.1.4 Paper labels

Two of the paper fields require special consideration: `topics` and `content_tags`. Since these labels were assigned manually, based primarily on human judgement, the data likely includes mistakes, omission, and bias, and should therefore be considered more critically than the other fields.

#### 2.1.4.1 Paper topics

more than one topic per paper; topics overlap in areas and are not exhaustive or precisely defined.

For the Topic column in Table 2.1 above, the most frequently ocurring topic was chosen to represent it, as described later in Sec. @ref{sec:topic-dist-by-conf}. Most topics have at least one conference wherein they ocurred most, although multi-topic papers and conferences are common. Each topic also has a single color associated with it throughout this document, as a visual aid in graphical representations. The complete list of topic tags, as well as their associated colors, can be found in Table 2.3. A later chapter (Ch. **??**) explores the properties and distributions of these topics.

Table 2.3: Paper topics: each paper was associated with any number of these tags

| Tag | Areas |
| --- | --- |
| Architecture | Computer architecture |
| Benchmark | Workloads, bechnmarking, metrics, and methodology |
| Cloud | Cloud computing and related infrastructure |
| Compilers | Compilers, language and debugging sopport, runtime systems |
| Concurrency | Parallel and distributed computing |
| Data | Big data applications and infrastructure |
| DB | Databases, key-value stores, and DBMS |
| Energy | Power and energy efficiency |
| GPGPU | Accelerator technologies |
| HPC | High performance computing and supercomputing applications |
| Network | Networking algorithms and protocols, wireless networks, switching and routing |
| OS | Operating systems, scheduling, resource management |
| Security | Security, privacy, encryption, resilience |
| Storage | Persistent storage: disk, flash, ram, and emerging technologies |
| VM | Virtual machines, networks, and containers |

#### 2.1.4.2 Content tags

Content tags for paper: One or more of:
List of topics in Table 2.4.

Full discussion in Ch. **??**.

Table 2.4: Content tags for systems papers

| Tag | Description |
| --- | --- |
| system | Reports design, implementation, or performance of a newly engineered system. |
| positive | Reports new positive results (improvement over past work). |
| negative | Reports new negative results or failure to reproduce past results. Examples include a system that doesn’t yield the expected results, or workloads under which the proposed system performs worse than before. |
| experience | Focuses on a new experience with a (possibly extant) system. Most systems papers describe experiences, but this tag is assigned only to papers where the experience is described in detail and consists of a top contribution for the paper. |
| data | Exposes or analyzes a dataset that hadn’t been previously discussed. |
| survey | Relies primarily on reviewing, or summarizing, previous work (literature or software). |
| position | Promotes opinions, optionally backed by new evidence. Positions represent suggestions or point of view that may be argued even based on the same data. |
| reproduction | Attempts to reproduce or debunk a previous result. A mere comparison to past results or systems does not constitute a reproduction. |
| open | Releases code or data openly set as part of the publication. |
| experiment | Reports results that were obtained from measuring a representative hardware or software system. An experimental result includes all hidden assumptions as well (as distinguished from a simulation, wherein all assumptions are coded). |
| simulation | Reports results that were obtained from simulating or emulating a system, for examle using a hardware simulator or a workload generator. |
| analysis | Reports results that were derived analytically, possibly with proofs. |
| preliminary | Describes a system or idea that isn’t fully completed or evaluated yet. |
| continuing | Describes a system (by the same authors) previously described in another paper. |
| bigscale | Describes experience on experiment on a uniquely large-scale system or software, one not easily reproducible by other research groups. |

## 2.2 Person data

Each person among a conference’s authors and PC members was looked up on Google Scholar and recorded near the time of publication (the postdate).

The JSON files in the `data/authors/` directory hold author information about each author and PC member, with one file per conference (the filename being the short conference name). Authors in multiple conferences appear in each conference’s file, but possibly with data more current for that postdate. All author metrics correspond to the latest data in the record (last *citedby* date).

These author records were derived from the person’s Google Scholar (GS) profile using `src/extract_authors.py` and manual editing. Authors that couldn’t be uniquely identified on GS have a single empty string instead of a record. The rest hold the following items (empty fields, where no data was available or applicable) are denoted as empty strings or lists, according to the field’s type):

### Field description

- `name` (string): Person’s name (also used as the key to each author record)
- `affiliation` (string): Person’s institute, if included in the conference data file in parenthesis (not necessarily reflecting the GS affiliation).
- `citedby` (map from date to int): For each date recorded, the total number of paper citations on GS.
- `gs_email` (string): Email institution from GS, starting with ‘@’ (such as `@csail.mit.edu`), or empty string if they haven’t entered any.
- `hindex` (int): H-index of person (see https://en.wikipedia.org/wiki/H-index).
- `hindex5y` (int): H-index in the past 5 years.
- `i10index` (int): Number of papers with 10 or more citations (see http://guides.library.cornell.edu/c.php?g=32272&p=203393).
- `i10index5y` (int): I-10 index in the past 5 years.
- `interests` (list of strings): List of free-form interests, as filled in by the author in their GS profile.
- `npubs` (int): Total number of publications on GS. This metric wasn’t measured reliabily, often under-reporting the actual GS data.

Obtaining this data from Google Scholar presented several challenges:

First, not all people actually maintain a GS profile (“NA” records). Fortunately, A large number do actually maintain such a profile. Out of 8196 unique authors in the data files, 5231 (63.82%) had an identifiable GS profile. The rate of profiles for the 3286 unique PC members and chairs (incuding some authors) is even higher, with 2762 GS profiles (84.05%).

Another difficult challenge is disambiguating multiple GS profiles for the same name (but different persons). These duplicates were handled manually (by examining the affiliation, publication record, and interests). In some cases, GS itself was unable to disambiguate persons successfully, leading to authors with many aggregated papers in their profile written by their doppelgangers. These profiles were omitted, rather than overestimating their metrics.

Last, some author names appeared in multiple variants, sometimes even in the same conference (such as ‘Andy’ vs. ‘Andrew’), leading to redundant records. Those also had to be identified and de-duped manually, with the help of a script (`src/gather_persons.py`) that searched for similar last names and/or affiliations.

## 2.3 Paper data

In addition to the static paper data in the conference file, the following data was collected at different dates per paper, and summarized in JSON files under the `data/papers/` directory (one file per conference):

- Key: same as in the `data/conf/` data, and used to key into JSON dictionary.
- Cited-by: for any given measurement date, how many papers cited this paper, if found on GS.
- Sprint-found: When was the first date detected (if any) that GS has a digital copy of the paper available for download.
- ID: the GS id for the paper, for repeated searches.
- Paper-found: The first date where a valid GS entry for this paper was detected.
- Title: The GS-reported title for the paper. May differ slightly from the one published by the conference homepage.
- URL: The link to a digital copy of the paper, if found.

## 2.4 Geographical data

The `data/geo` directory contains imported input files from various Web sources to compose
geographical data. The files included are:

- `countries.json` (from https://github.com/mledoze/countries): Country name (common), code, region and subregion, central lat/long coordinates, official languages, and land area (retrieved 2018-01-02).
  - `weoreptc.aspx` (from the IMF’s http://www.imf.org/external/pubs/ft/weo/2017/02/weodata/download.aspx: 2016 nominal gross domestic product (retrieved 2018-01-02).
  - `WPP2017_MIGR_F01_NET_MIGRATION_RATE.xlsx` (from the UN DESA’s https://esa.un.org/unpd/wpp/Download/Standard/Population/): 2015 net migration rate (retrieved 2018-01-02).
- `WPP2017_POP_F01_1_TOTAL_POPULATION_BOTH_SEXES.xlsx` (from the UN DESA’s https://esa.un.org/unpd/wpp/Download/Standard/Population/): 2015 total population size (retrieved 2018-01-02).
- `WPP2017_POP_F02_POPULATION_GROWTH_RATE.xlsx` (from the UN DESA’s https://esa.un.org/unpd/wpp/Download/Standard/Population/: 2015 population growth rate (retrieved 2018-01-02).
- `WPP2017_POP_F04_SEX_RATIO_OF_TOTAL_POPULATION.xlsx` (from the UN DESA’s https://esa.un.org/unpd/wpp/Download/Standard/Population/): 2015 ratio of males to females (retrieved 2018-01-02).
- `WPP2017_POP_F05_MEDIAN_AGE.xlsx` (from the UN DESA’s https://esa.un.org/unpd/wpp/Download/Standard/Population/): 2015 median population age (retrieved 2018-01-02).

## 2.5 Challenges

Sometimes, the hardest part of a data analysis is the collection and cleaning of the input data, before even writing a single line of analysis code. This data set is no exception, and various factors contributed to either missing, noisy, or plain erroneous data. The top two challenges in collecting this data were disambiguating author names–matching them to a single GS profile correctly, and obtaining detailed conference information in the presence of partial and non-standard proceedings and web sites.

### 2.5.1 Defining “Systems”

The actual definition of the field of computer systems has immediate consequences for the conference selection and data analysis, and yet cannot be done precisely. Although most computer scientists may have an intuitive understanding of what areas belong to the field, a widely accepted and precise definition of the field and its sub-topics remains elusive. Even the definition in the introduction chapter remains ambiguous, open-ended, and non-exclusive. It is also likely to change over time, as peripheral fields and interdisciplinary papers shift in and out of traditional systems conferences. For example, should a paper on a linear-algebra machine-learning algorithm to predict weather be treated as a systems paper if it’s also implemented on a supercopmuter in a way that minimizes energy use and maximizes scalability?

In the absence of an objective and unobjectionable definition, we’re left with the vague defintion in Ch. 1 and subjective judgements. These come into play primarily in two types of data decisions:

1. Which conferences should be included in our data set? Some of the fifty systems conferences are easy to identify as such, because they’re narrowly focused on an uncontroversial sub-field of systems, such as ISCA in architecture. Other conferences include papers that are more theoretical or interdisciplinary, and may legitimately be judged as “non-systems” by its practitioners. And others, like our six “control” conferences, contain a mix of topics that are certainly related to systems and may employ similar research methodologies, even if not widely considered a systems topic. This study is therefore open to critism and interpretation based on the choice of “systems” conference definition. Since all of its data and source code is openly available, the reader is encouraged to try re-running the analysis on their own subsets of conferences and see if they reach different conclusions.
2. What are the subfields of computer systems? As previously described in Sec. 2.1.4.1, each paper was assigned zero or more topic tags from a set of 3829 sub-topics of computer systems. These topics were arbitrarily chosen, based on the author’s experience with computer systems. But just like “computer systems”, their definitions are also crippled by vagueness, non-exclusivity, subjective judgement, and lack of consensus. Many of these tags describe concepts that are not necessarily even exclusive to computer systems, such as the “Bencmark” tag, which describes research focused on the methodology and measurement of computer systems, but could easily apply to other fields as well. Again, recognizing that these choices are subjective and somewhat arbitrary, readers are encouraged to try their own definitions.

### 2.5.2 Author disambiguation

Many names are common enough to have multiple GS profiles associated with them (sometimes even from the same institute or research field!). Not all conferences list the affiliation of authors, and sometimes not even their full name. Some authors had different spellings of their name (or typos), and some had contractions or initials used inconsistently. And in some cases, authors had a unique name with its own single GS profile, but one that belonged, or was mixed with, a different researcher’s from a completely different field.

Disambiguating author names is a well-known and difficult problem [3]. Certainly proposal like assigning each author with a unique ID [2] could help in the future. But for this data set, disambiguation took place as a painstakingly manual process of verifying each name conflict and each misaligned GS profile. For every potential ambiguity, I verified the correct GS profile, for example, by comparing the author’s publication list to the paper(s) in this data set, looking for recently changed affiliation, or comparing email addresses. In cases where ambiguity couldn’t be confidently resolved, or when an author’s GS profile appeared to be conflated by more than one actual authors, I simply dropped their GS information from the data set. In addition, the author aggregation script (`src/gather_persons.py`) checks for similar and duplicate names and warns where ambiguity is possible.

### 2.5.3 Partial conference data

Not all proceedings of all conferences include all the data fields collected, such as the total number of reviews written (in fact, most didn’t include this particular field). Some conferences had minor conflicts between the online program and the proceedings’ list of papers (either in title, author list, or even paper count). In a few cases, web pages for conferences became unavailable after the conference concluded. To resolve these inconsistencies, and augment the missing data, I emailed the program committee chairs and requested the missing information. I thank all the chairs that responded to my queries.

### Bibliography

[2] Haak, L.L., Fenner, M., Paglione, L., Pentz, E. and Ratner, H. 2012. ORCID: A system to uniquely identify researchers. *Learned Publishing*. 25, 4 (2012), 259–264.

[3] Han, H., Giles, L., Zha, H., Li, C. and Tsioutsiouliklis, K. 2004. Two supervised learning approaches for name disambiguation in author citations. *Proceedings of the 2004 joint ACM/IEEE conference on digital libraries* (2004), 296–305.

---

1. The PDF versions of the papers are excluded from the accompanying data set because of size and copyright constraints.↩
