# Supplementary figures and images for: Research artifacts and citations in computer systems papers
Source: PeerJ Comput Sci. 2022 Feb 7;8:e887. doi: 10.7717/peerj-cs.887 (PMC9044204; doi:10.7717/peerj-cs.887)

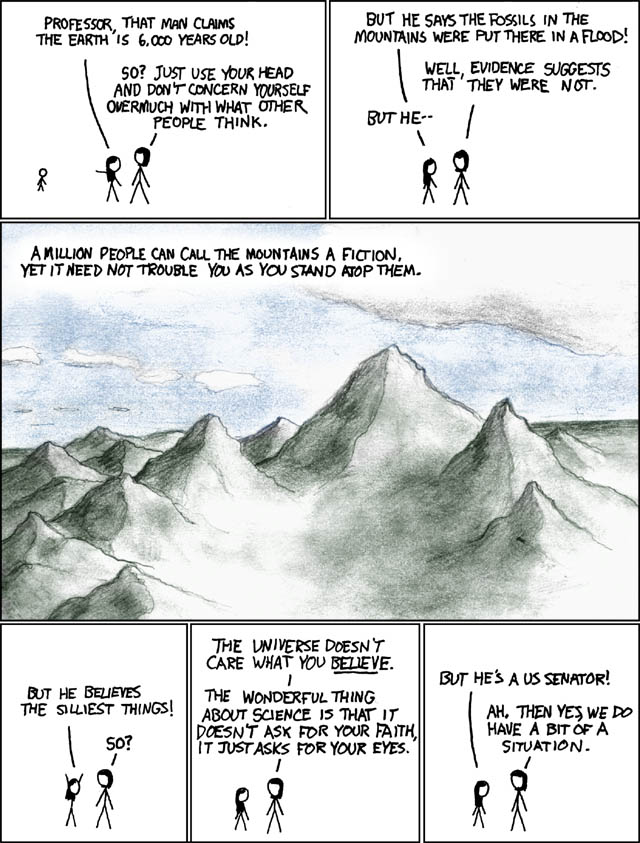

Supplement: Supplemental Information 2 — Please refer to all *.md files for detailed data descriptions. [file peerj-cs-08-887-s002.tbz2 › sysconf/docs/images/beliefs.jpg]

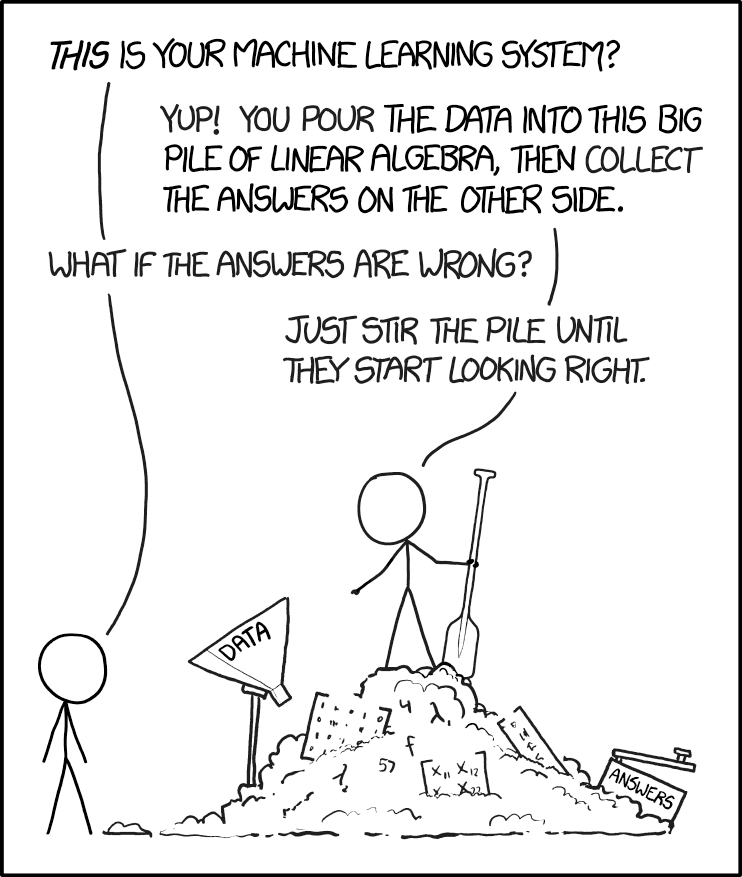

Supplement: Supplemental Information 2 — Please refer to all *.md files for detailed data descriptions. [file peerj-cs-08-887-s002.tbz2 › sysconf/docs/images/machine_learning_2x.png]

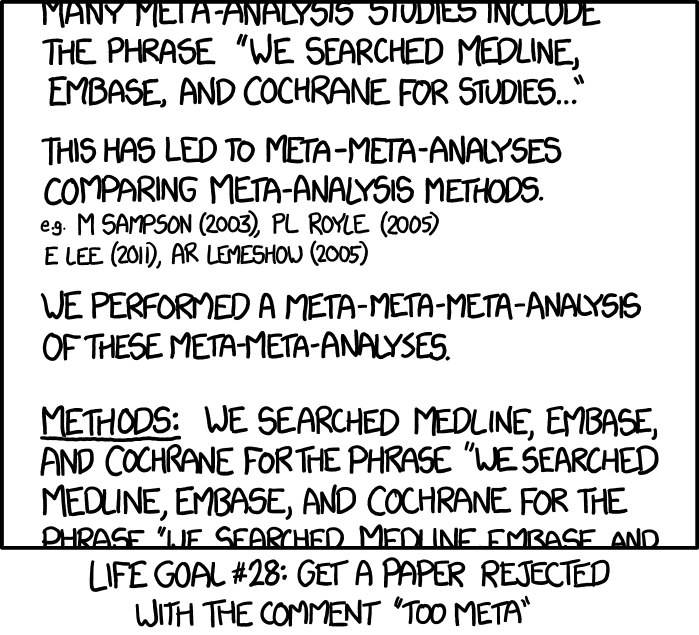

Supplement: Supplemental Information 2 — Please refer to all *.md files for detailed data descriptions. [file peerj-cs-08-887-s002.tbz2 › sysconf/pubs/web/images/meta-analysis_2x.png]

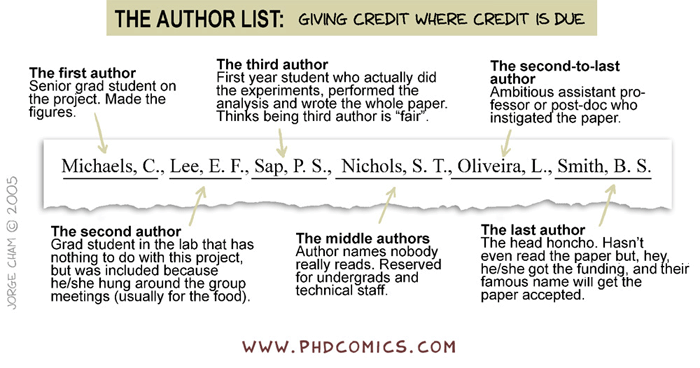

Supplement: Supplemental Information 2 — Please refer to all *.md files for detailed data descriptions. [file peerj-cs-08-887-s002.tbz2 › sysconf/pubs/web/images/phd031305s.gif]

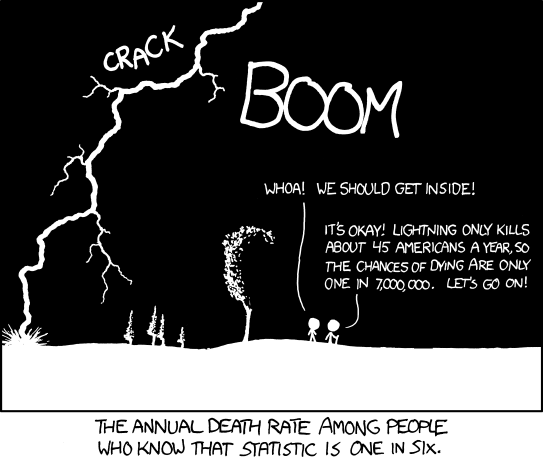

Supplement: Supplemental Information 2 — Please refer to all *.md files for detailed data descriptions. [file peerj-cs-08-887-s002.tbz2 › sysconf/pubs/web/images/conditional_risk.png]

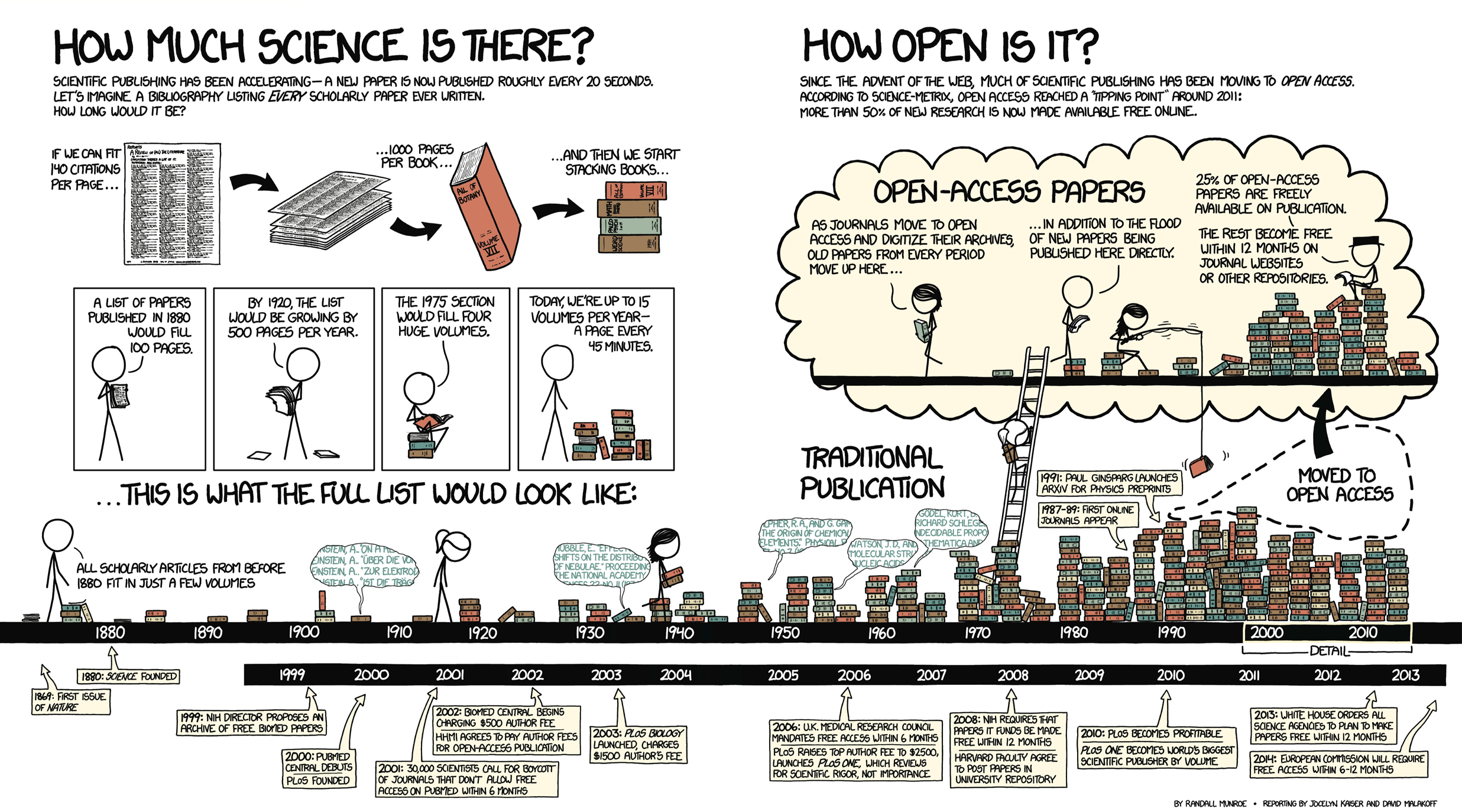

Supplement: Supplemental Information 2 — Please refer to all *.md files for detailed data descriptions. [file peerj-cs-08-887-s002.tbz2 › sysconf/pubs/web/images/infographic.jpg]

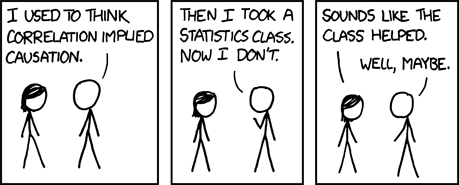

Supplement: Supplemental Information 2 — Please refer to all *.md files for detailed data descriptions. [file peerj-cs-08-887-s002.tbz2 › sysconf/pubs/web/images/correlation.png]

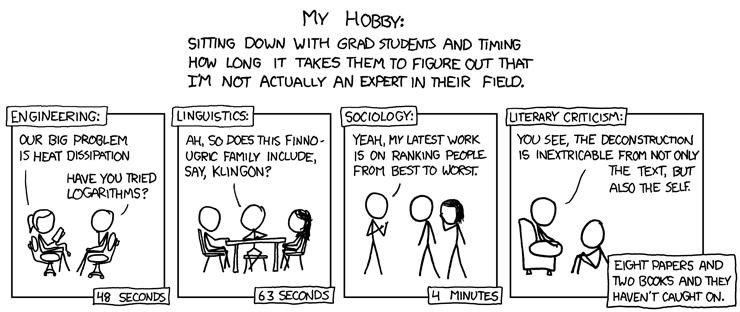

Supplement: Supplemental Information 2 — Please refer to all *.md files for detailed data descriptions. [file peerj-cs-08-887-s002.tbz2 › sysconf/pubs/web/images/impostor.png]

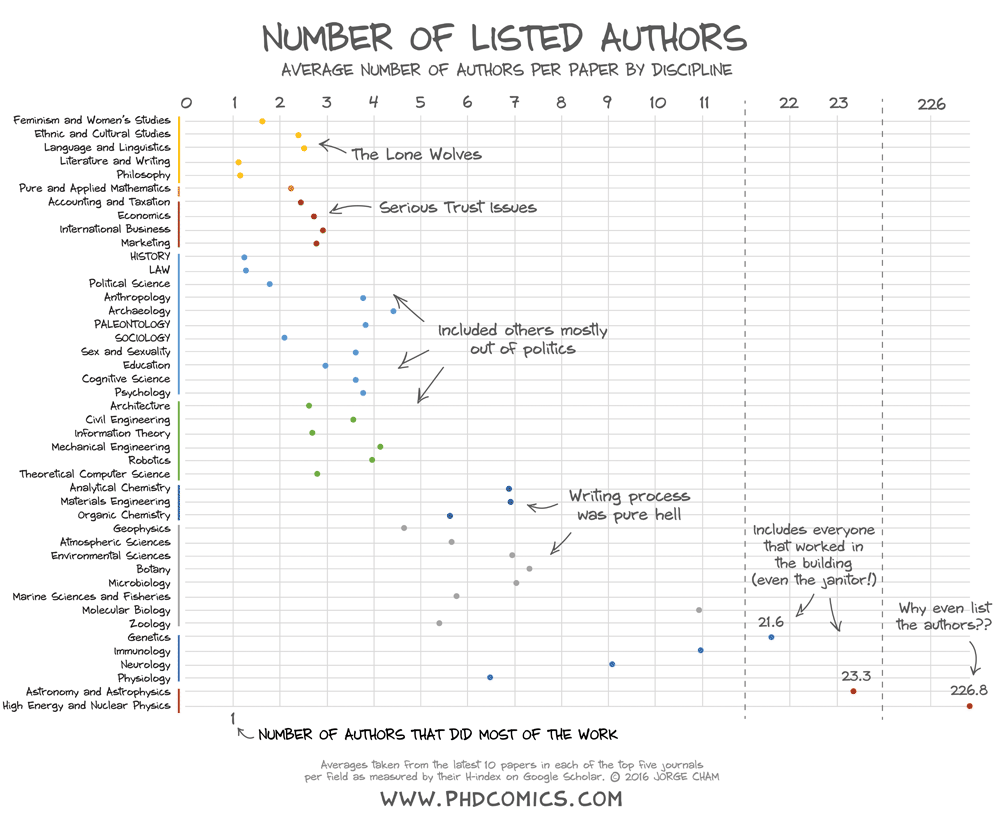

Supplement: Supplemental Information 2 — Please refer to all *.md files for detailed data descriptions. [file peerj-cs-08-887-s002.tbz2 › sysconf/pubs/web/images/phd120916s.gif]

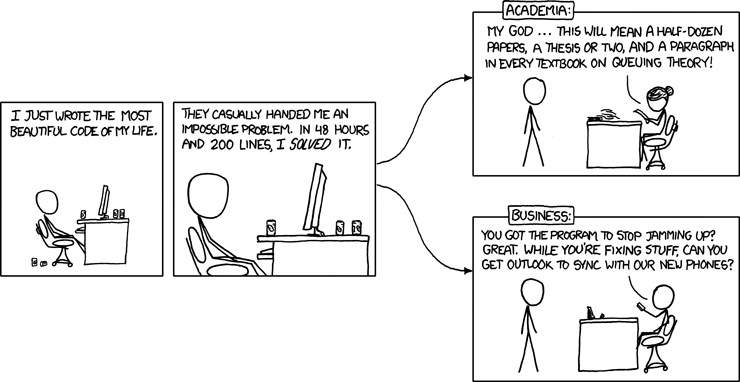

Supplement: Supplemental Information 2 — Please refer to all *.md files for detailed data descriptions. [file peerj-cs-08-887-s002.tbz2 › sysconf/pubs/web/images/academia_vs_business.png]

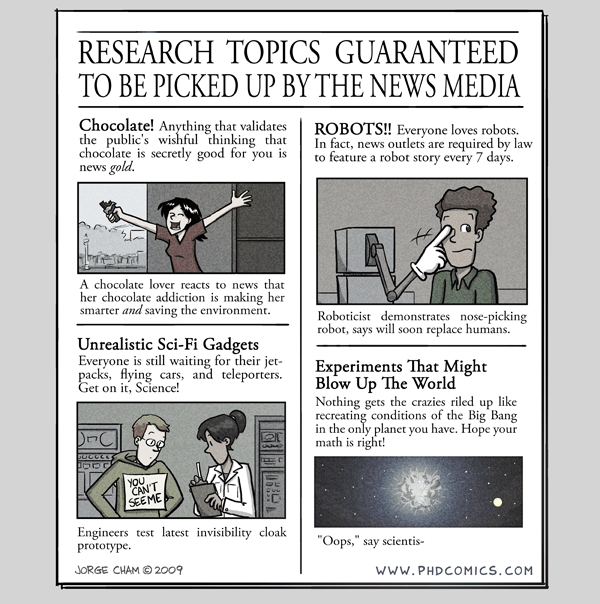

Supplement: Supplemental Information 2 — Please refer to all *.md files for detailed data descriptions. [file peerj-cs-08-887-s002.tbz2 › sysconf/pubs/web/images/phd052009s.gif]

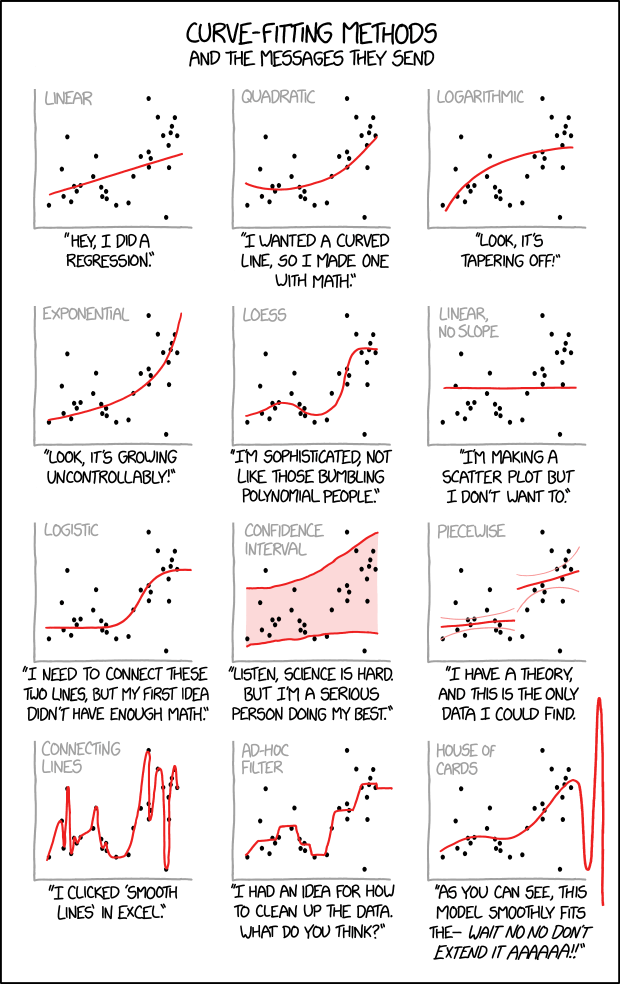

Supplement: Supplemental Information 2 — Please refer to all *.md files for detailed data descriptions. [file peerj-cs-08-887-s002.tbz2 › sysconf/pubs/web/images/curve_fitting.png]

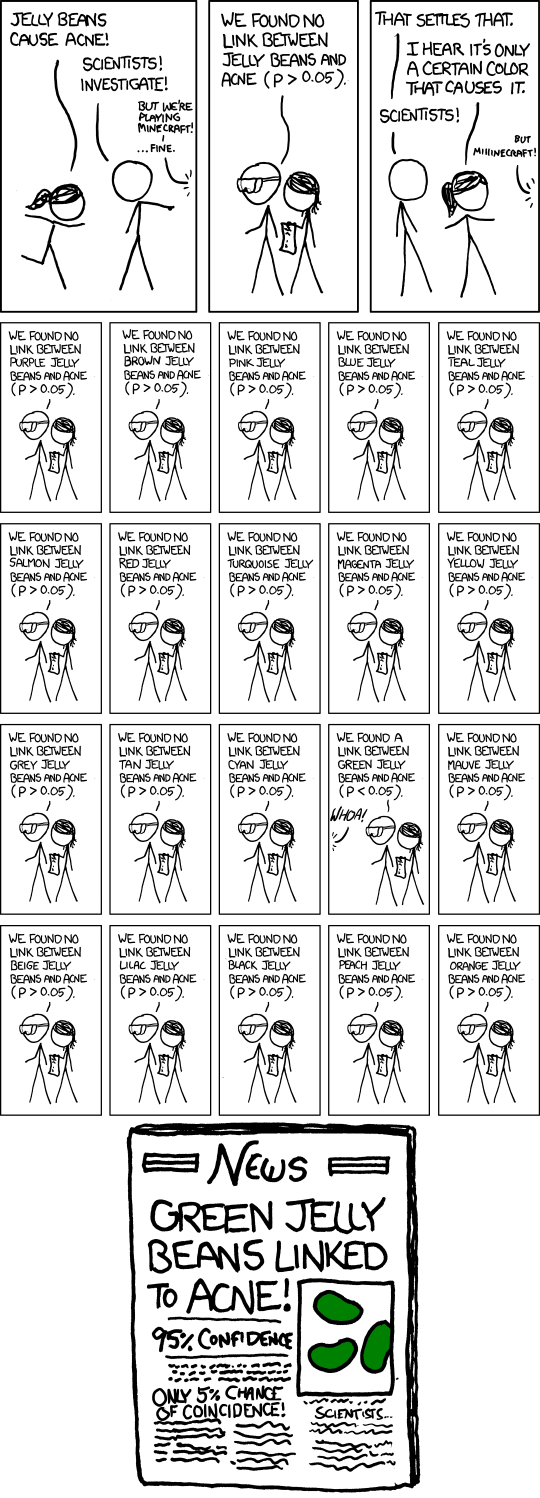

Supplement: Supplemental Information 2 — Please refer to all *.md files for detailed data descriptions. [file peerj-cs-08-887-s002.tbz2 › sysconf/pubs/web/images/significant.png]

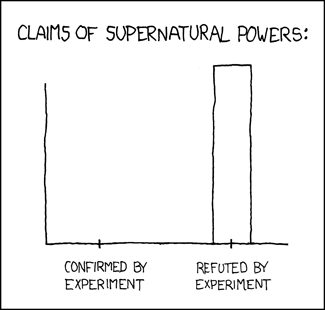

Supplement: Supplemental Information 2 — Please refer to all *.md files for detailed data descriptions. [file peerj-cs-08-887-s002.tbz2 › sysconf/pubs/web/images/the_data_so_far.png]

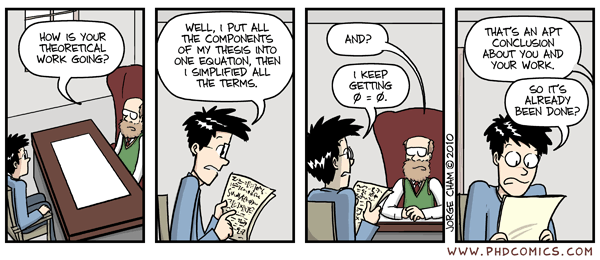

Supplement: Supplemental Information 2 — Please refer to all *.md files for detailed data descriptions. [file peerj-cs-08-887-s002.tbz2 › sysconf/pubs/web/images/phd110110s.gif]

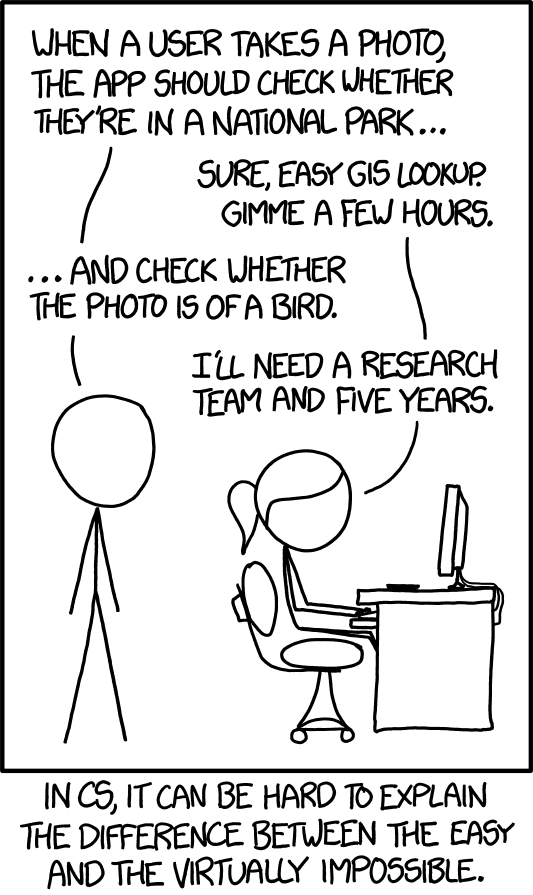

Supplement: Supplemental Information 2 — Please refer to all *.md files for detailed data descriptions. [file peerj-cs-08-887-s002.tbz2 › sysconf/pubs/web/images/tasks_2x.png]

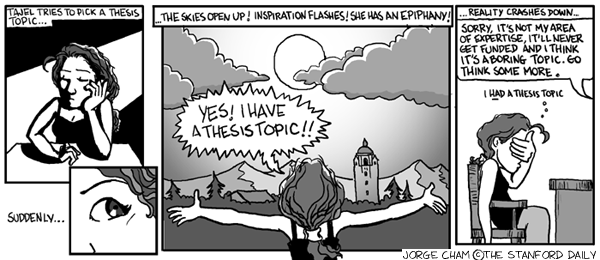

Supplement: Supplemental Information 2 — Please refer to all *.md files for detailed data descriptions. [file peerj-cs-08-887-s002.tbz2 › sysconf/pubs/web/images/phd100998s.gif]

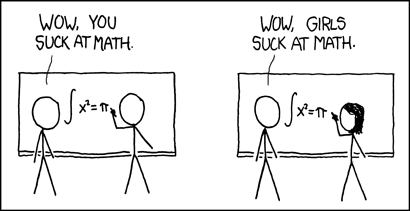

Supplement: Supplemental Information 2 — Please refer to all *.md files for detailed data descriptions. [file peerj-cs-08-887-s002.tbz2 › sysconf/pubs/web/images/how_it_works.png]

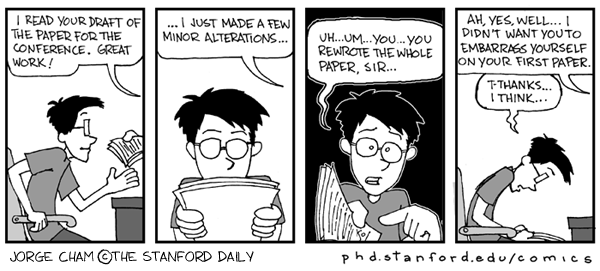

Supplement: Supplemental Information 2 — Please refer to all *.md files for detailed data descriptions. [file peerj-cs-08-887-s002.tbz2 › sysconf/pubs/web/images/phd010500s.gif]

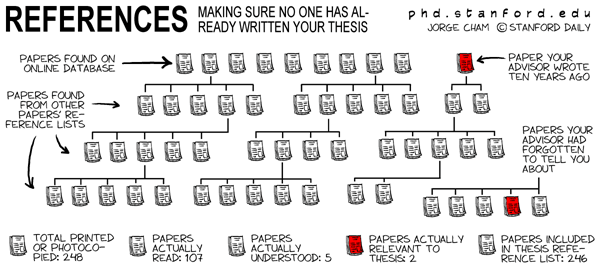

Supplement: Supplemental Information 2 — Please refer to all *.md files for detailed data descriptions. [file peerj-cs-08-887-s002.tbz2 › sysconf/pubs/web/images/phd022702s.gif]

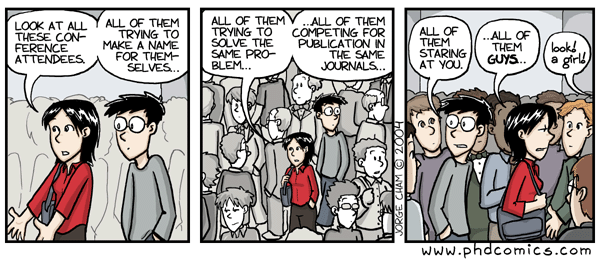

Supplement: Supplemental Information 2 — Please refer to all *.md files for detailed data descriptions. [file peerj-cs-08-887-s002.tbz2 › sysconf/pubs/web/images/phd081604s.gif]

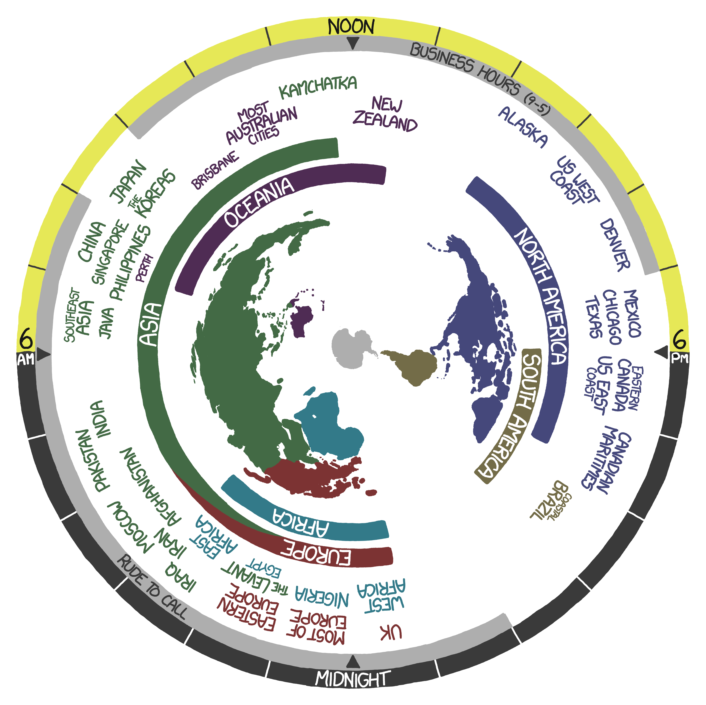

Supplement: Supplemental Information 2 — Please refer to all *.md files for detailed data descriptions. [file peerj-cs-08-887-s002.tbz2 › sysconf/pubs/web/images/11h30m.png]
